# The proprotein convertase subtilisin/kexin type 9 gene E670G polymorphism and serum lipid levels in the Guangxi Bai Ku Yao and Han populations

**DOI:** 10.1186/1476-511X-10-5

**Published:** 2011-01-13

**Authors:** Lynn Htet Htet Aung, Rui-Xing Yin, Lin Miao, Xi-Jiang Hu, Ting-Ting Yan, Xiao-Li Cao, Dong-Feng Wu, Qing Li, Shang-Ling Pan, Jin-Zhen Wu

**Affiliations:** 1 Department of Cardiology, Institute of Cardiovascular Diseases, the First Affiliated Hospital, Guangxi Medical University, 22 Shuangyong Road, Nanning 530021, Guangxi, People's Republic of China; 2 Department of Pathophysiology, School of Premedical Sciences, Guangxi Medical University, Nanning 530021, Guangxi, People's Republic of China

## Abstract

**Background:**

Proprotein convertase subtilisin-like kexin type 9 (PCSK9) plays a key role in regulating plasma low-density lipoprotein cholesterol (LDL-C) levels. However, the association of E670G (rs505151) polymorphism in the PCSK9 gene and serum lipid levels is inconsistent in several previous studies. The present study was undertaken to detect the association of PCSK9 E670G polymorphism and several environmental factors with serum lipid levels in the Guangxi Bai Ku Yao and Han populations.

**Methods:**

A total of 649 subjects of Bai Ku Yao and 646 participants of Han were randomly selected from our previous samples. Genotypes of the PCSK9 E670G polymorphism were determined via polymerase chain reaction and restriction fragment length polymorphism combined with gel electrophoresis, and then confirmed by direct sequencing.

**Results:**

Serum levels of total cholesterol, high-density lipoprotein cholesterol (HDL-C), LDL-C, and apolipoprotein (Apo) AI were lower in Bai Ku Yao than in Han (*P *< 0.01 for all). The frequency of G allele was 2.00% in Bai Ku Yao and 4.80% in Han (*P *< 0.01). There was significant difference in the genotypic and allelic frequencies between Bai Ku Yao and Han (*P *< 0.01); between normal LDL-C (≤ 3.20 mmol/L) and high LDL-C subgroups (> 3.20 mmol/L, *P *< 0.01) in Bai Ku Yao; and between normal HDL-C (≥ 0.91 mmol/L) and low HDL-C (< 0.91 mmol/L, *P *< 0.05), between normal ApoAI (≥ 1.00 g/L) and low ApoAI (< 1.00 g/L, *P *< 0.05), or between normal ApoAI/ApoB ratio (≥ 1.00) and low ApoAI/ApoB ratio (< 1.00, *P *< 0.01) subgroups in Han. The G allele carriers in Han had higher serum HDL-C levels and the ratio of ApoAI to ApoB than the G allele noncarriers. The G allele carriers in Han had higher serum HDL-C and ApoAI levels than the G allele noncarriers in males (*P *< 0.05 for each), whereas the G allele carriers had lower serum ApoB levels and higher the ratio of ApoAI to ApoB than the G allele noncarriers in females (*P *< 0.05 for all). Serum HDL-C and ApoAI levels in Han were correlated with genotypes (*P *< 0.05) in males, and serum ApoB levels and the ratio of ApoAI to ApoB were associated with genotypes (*P *< 0.05) in females.

**Conclusions:**

The PCSK9 E670G polymorphism is mainly associated with some serum lipid parameters in the Han population. The G allele carriers had higher serum HDL-C and ApoAI levels in males, and lower serum ApoB levels and higher the ApoAI/ApoB ratio in females than the G allele noncarriers.

## Introduction

Abnormalities in lipid metabolism such as elevated serum levels of total cholesterol (TC) [1], triglyceride (TG) [2], low-density lipoprotein cholesterol (LDL-C) [3], and apolipoprotein (Apo) B [4], or low levels of high-density lipoprotein cholesterol (HDL-C) and ApoAI [4-6] are considered as major risk factors for coronary artery disease (CAD). It is generally agreed that dyslipidemia is complex and the result of the interactions [7,8] of multiple genes [9-11] and multiple environmental factors [12,13]. Both family and twin studies have consistently shown that 40-60% of the interindividual variation in plasma lipid phenotypes is genetic in etiology [14-16]. Although multiple genetic defects have been identified that cause rare Mendelian forms of severe hypercholesterolemia or hypocholesterolemia, the sequence variations in the genome accounting for most of the variation in serum lipid levels in the general population have not been determined.

Proprotein convertase subtilisin-like kexin type 9 (PCSK9, OMIM 607786) is a newly discovered serine protease that plays a key role in LDL-C homeostasis by mediating LDL receptor (LDLR) breakdown through a post-transcriptional mechanism [17-20]. PCSK9 may also regulate ApoB-containing lipoprotein production and ApoB secretion [21,22], and promote production of nascent very low-density lipoprotein (VLDL) in the fasting state [23]. Human PCSK9 gene is approximately 22 kb long, comprising the promoter region and 12 exons, and it is located on chromosome 1p32. The gene produces a mRNA of 3636 bp encoding a 692-amino acid glycoprotein. This protein, also called neural apoptosis regulated convertase, is a serine protease belonging to the protease K subfamily of subtilases. It is a subfamily of proteases largely involved in the processing of inactive precursor proteins to the active product and seems to be involved in the inactivation and degradation of LDLR [24-26]. PCSK9 contains a signal sequence and prodomain at its N-terminus, followed by a catalytic domain and cysteine-rich carboxy-terminal domain. PCSK9 undergoes autocatalytic cleavage in the endoplasmic reticulum, releasing the aminoterminal prodomain. The prodomain remains associated with the processed form of PCSK9 as it transits through the secretory pathway. Adenoviral-mediated over-expression of human PCSK9 in mice promotes the accumulation of LDL-C in the plasma but this response is absent in LDLR-deficient animals [18,20,27]. Recent studies show that PCSK9 binds directly to the extracellular domain of the LDLR [28,29] and increases its degradation [28]. PCSK9 is expressed most abundantly in the liver, kidney, and small intestine [30]. PCSK9 may enhance degradation of other receptor types or proteins during the development of cerebellum and telencephalon [30] and promote cerebellar cortical neurogenesis, possibly by increased recruitment of undifferentiated neural progenitor cells into the neuronal lineage [31]. The characterization of 'gain-of-function' versus 'loss-of-function' alleles of PCSK9 is based on the phenotype (LDL-C), and not on defined biochemical alterations. Several studies have found that missense mutations increasing the activity of PCSK9 (i.e., gain-of-function mutations) result in an increase of LDL-C levels and CAD [22,32-34] whereas nonsense mutations reducing PCSK9 activity (i.e., loss-of-function mutations) have the opposite effect, lowering LDL-C levels and reducing risk of CAD [35-37]. These findings reveal that PCSK9 activity is a major determinant of plasma levels of LDL-C in humans and make it an attractive therapeutic target for LDL-C lowering. Among the genetic variants of the PCSK9 gene, a common single nucleotide polymorphism (SNP), E670G (rs505151), in exon 12 deserved greater scrutiny, as it was responsible for an amino acid change that could potentially be associated with altered PCSK9 activity. The E670G polymorphism in humans has been found to be associated with modifications of serum LDL-C levels in some studies [38-42] but not in others [37,43-45].

China is a multiethnic country. There are 56 ethnic groups. Han is the largest ethnic group and Yao is the eleventh largest minority among the 55 minority groups according to the population size. There is also an isolated branch of the Yao minority, Bai Ku Yao (White-trouser Yao, all of men wear white knee-length knickerbockers). The population size is about 30000. Because of isolation from the other ethnic groups, the special customs and cultures including their clothing, intra-ethnic marriages, ballad, funeral, bronze drum, spinning top, dietary habits, and corn wine and rum intakes are still completely preserved to the present day. Previous reports by our group have found that several serum lipid phenotypes were lower in Bai Ku Yao than in Han Chinese from the same villages [12,13]. This ethnic difference in serum lipid profiles is still not well known. We hypothesized that some genetic polymorphisms may be different between the two ethnic groups [7-11]. Thus, the aim of this study was to detect the association of PCSK9 E670G polymorphism and several environmental factors with serum lipid phenotypes in the Guangxi Bai Ku Yao and Han populations.

## Materials and methods

### Study population

The study population included 649 subjects of Bai Ku Yao who reside in Lihu and Baxu villages in Nandan County, Guangxi Zhuang Autonomous Region, People's Republic of China. They were randomly selected from our previous stratified randomized cluster samples [12,13]. The ages of the subjects ranged from 15 to 80 years, with an average age of 35.68 ± 13.69 years. There were 324 males (49.92%) and 325 females (50.08%). All subjects were rural agricultural workers. The subjects accounted for 2.16% of total Bai Ku Yao population. During the same period, a total of 646 people of Han Chinese who reside in the same villages were also randomly selected from our previous stratified randomized cluster samples [12,13]. The average age of the subjects was 36.59 ± 17.56 years (range 15 to 80). There were 320 men (49.54%) and 326 women (50.46%). All of them were also rural agricultural workers. All study subjects were essentially healthy and had no evidence of any chronic illness, including hepatic, renal, or thyroid. The participants with a history of heart attack or myocardial infarction, stroke, congestive heart failure, diabetes or fasting blood glucose ≥ 7.0 mmol/L determined by glucose meter were not included. The participants were not taking medications known to affect serum lipid levels (lipid-lowering drugs such as statins or fibrates, beta-blockers, diuretics, or hormones) at the time of sample extraction. The present study was approved by the Ethics Committee of the First Affiliated Hospital, Guangxi Medical University. Informed consent was obtained from all subjects after they received a full explanation of the study.

### Epidemiological survey

The survey was carried out using internationally standardized methods, following a common protocol [46]. Demographic data, socioeconomic status, and lifestyle factors were collected with standardized questionnaires. The alcohol information included questions about the number of liangs (about 50 g) of rice wine, corn wine, rum, beer, or liquor consumed during the preceding 12 months. Alcohol consumption was categorized into groups of grams of alcohol per day: < 25 and ≥ 25. Smoking status was categorized into groups of cigarettes per day: < 20 and ≥ 20. At each examination, weight and height were measured with subjects in light clothing and without shoes. Body weight was measured to the nearest 50 grams, using a portable balance scale. Height was measured to the nearest 0.5 cm with a portable steel measuring device. Body mass index (BMI) was calculated as the weight in kilograms divided by the square of height in meters. Sitting blood pressure was measured three times with the use of a mercury sphygmomanometer after the subjects had a 5-minute rest, and the average of the three measurements was used for the level of blood pressure. Systolic blood pressure was determined by the first Korotkoff sound, and diastolic blood pressure by the fifth Korotkoff sound.

### Biochemical measurements

Fasting venous blood samples (8 mL) were obtained from all participants between 8 and 11 AM. A part of the sample (3 mL) was collected into glass tubes and allowed to clot at room temperature, and used to determine serum lipid levels. Another part of the sample (5 mL) was transferred to tubes with anticoagulate solution (4.80 g/L citric acid, 14.70 g/L glucose, and 13.20 g/L tri-sodium citrate) and used to extract DNA. Immediately following clotting serum was separated by centrifugation for 15 minutes at 3000 rpm. The levels of TC, TG, HDL-C, and LDL-C in samples were measured using commercial enzymatic reagents, Tcho-1, TG-LH (RANDOX Laboratories Ltd., Ardmore, Diamond Road, Crumlin Co. Antrim, United Kingdom, BT29 4QY), Cholestest N HDL, and Cholestest LDL (Daiichi Pure Chemicals Co., Ltd., Tokyo, Japan); respectively. Serum ApoAI and ApoB levels were detected by the immunoturbidimetric immunoassay using a commercial kit (RANDOX Laboratories Ltd.). All determinations were performed with an autoanalyzer (Type 7170A; Hitachi Ltd., Tokyo, Japan) in the Clinical Science Experiment Center of the First Affiliated Hospital, Guangxi Medical University [12,13].

### DNA amplification and genotyping

Genomic DNA was isolated from peripheral blood leukocytes using the phenol-chloroform method as previously described [7-11]. The extracted DNA was maintained at 4°C until analysis. Genotyping of the PCSK9 E670G polymorphism was performed by polymerase chain reaction and restriction fragment length polymorphism (PCR-RFLP). PCR amplification was performed using 5'-CACGGTTGTGTCCCAAATGG-3' and 5'-GAGAGGGACAAGTCGGAACC-3' (Sangon, Shanghai, People's Republic of China) as the forward and reverse primer pairs; respectively. Each amplification reaction was performed using 100 ng of genomic DNA in 25 μL of reaction mixture consisting of 25 μmol/L of each primer, 200 μmol/L of each deoxynucleotide triphoisphate, 2.5 μL of 10 × PCR buffer (100 mM Tris-HCl, pH 8.3, 500 mM KCl, 20 mM MgCl_2_, 1% Triton), and 2 units of *Taq *polymerase. After initial denaturizing at 94°C for 5 min, the reaction mixture was subjected to 35 cycles of 30 s denaturation at 94°C, 30 s annealing at 58°C and extension 30 s at 72°C, followed by a final 5 min extension at 72°C. After electrophoresis on a 1.2% agarose gel with 0.5 μg/mL ethidium bromide (EB), the amplification products were visualized under ultraviolet light. Then 2 U of *Eam *1104I restriction enzyme was added directly to the PCR products (10 μL) and digested at 37°C overnight. After restriction enzyme digestion of the amplified DNA, the genotypes were identified by electrophoresis on 2% agagarose gels and visualized with ethidium-bromide staining ultraviolet illumination. Genotypes were scored by an experienced reader blinded to epidemiological data and serum lipid levels. Six samples (AA, AG and GG genotypes in two; respectively) detected by the PCR-RFLP were also confirmed by direct sequencing. The PCR products were purified by low melting point gel electrophoresis and phenol extraction, and then the DNA sequences were analyzed in Shanghai Sangon Biological Engineering Technology & Services Co., Ltd., People's Republic of China.

### Diagnostic criteria

The normal values of serum TC, TG, HDL-C, LDL-C, ApoAI, ApoB levels, and the ratio of ApoAI to ApoB in our Clinical Science Experiment Center were 3.10-5.17, 0.56-1.70, 0.91-1.81, 2.70-3.20 mmol/L, 1.00-1.78, 0.63-1.14 g/L, and 1.00-2.50; respectively. The individuals with TC > 5.17 mmol/L and/or TG > 1.70 mmol/L were defined as hyperlipidemic [12,13]. Hypertension was diagnosed according to the criteria of 1999 World Health Organization-International Society of Hypertension Guidelines for the management of hypertension [47,48]. The diagnostic criteria of overweight and obesity were according to the Cooperative Meta-analysis Group of China Obesity Task Force. Normal weight, overweight and obesity were defined as a BMI < 24, 24-28, and > 28 kg/m^2^; respectively [49].

### Statistical analyses

Epidemiological data were recorded on a pre-designed form and managed with Excel software. Data analysis was performed using the statistical software package SPSS 13.0 (SPSS Inc., Chicago, Illinois). Quantitative variables were expressed as mean ± standard deviation (serum TG levels were presented as medians and interquartile ranges). Qualitative variables were expressed as percentages. Allele frequency was determined via direct counting, and the standard goodness-of-fit test was used to test the Hardy-Weinberg equilibrium. Difference in genotype distribution between the groups was obtained using the chi-square test. The difference in general characteristics between Bai Ku Yao and Han was tested by the Student's unpaired *t*-test. The association of genotypes and serum lipid parameters was tested by analysis of covariance (ANCOVA). Sex, age, BMI, blood pressure, alcohol consumption, cigarette smoking were adjusted for the statistical analysis. In order to assess the association of serum lipid levels with genotypes (AA = 1, AG/GG = 2) and several environment factors, multiple linear regression analysis with stepwise modeling was also performed in the combined population of Bai Ku Yao and Han, Bai Ku Yao, Han, males and females; respectively. A *P *value of less than 0.05 was considered statistically significant.

## Results

### General characteristics and serum lipid levels

The general characteristics and serum lipid levels between the Bai Ku Yao and Han populations are presented in Table 1. The levels of height, weight, systolic blood pressure, pulse pressure, serum TC, HDL-C, LDL-C, ApoAI were lower in Bai Ku Yao than in Han Chinese (*P *< 0.01-0.001), whereas the percentage of subjects who consumed alcohol was higher in Bai Ku Yao than in Han (*P *< 0.001). There were no significant differences in the levels of BMI, diastolic blood pressure, serum TG, ApoB, the ratio of ApoAI to ApoB, age structure, the percentage of subjects who smoked cigarettes, or the ratio of male to female between the two ethnic groups (*P *> 0.05 for all).

**Table 1:** Comparison of demographic, lifestyle characteristics and serum lipid levels between Bai Ku Yao and Han Chinese

Characteristic	Bai Ku Yao	Han Chinese	***t *(*χ***^**2**^**)**	*P*
Number	649	646	-	-
Male/female	324/325	320/326	0.139	0.889
Age (years)	35.68 ± 13.69	36.59 ± 17.56	-1.040	0.298
Height (cm)	153.27 ± 7.17	156.89 ± 8.83	-8.356	0.000
Weight (kg)	51.85 ± 6.99	54.32 ± 10.32	-5.044	0.000
Body mass index (kg/m^2^)	22.04 ± 2.28	22.03 ± 3.65	0.042	0.967
Systolic blood pressure (mmHg)	116.48 ± 15.78	119.54 ± 17.16	-3.339	0.001
Diastolic blood pressure (mmHg)	74.81 ± 8.99	75.34 ± 11.10	-0.936	0.350
Pulse pressure (mmHg)	41.66 ± 11.64	44.21 ± 11.9	-3.897	0.000
Cigarette smoking [n (%)]				
Nonsmoker	442 (68.1)	492 (76.2)		
< 20 cigarettes/day	101 (15.6)	46 (7.1)		
≥ 20 cigarettes/day	106 (2.3)	108 (16.7)	1.468	0.142
Alcohol consumption [n (%)]				
Nondrinker	383 (59.0)	490 (75.9)		
< 25 g/day	186 (28.7)	112 (17.3)		
≥ 25 g/day	74 (11.4)	44 (6.8)	5.653	0.000
Total cholesterol (mmol/L)	4.23 ± 0.93	4.63 ± 1.07	6.342	0.000
Triglyceride (mmol/L)	0.99 (0.66)	1.02 (0.74)	-1.716	0.086
HDL-C (mmol/L)	1.64 ± 0.41	1.75 ± 0.47	-4.643	0.000
LDL-C (mmol/L)	2.50 ± 0.76	2.65 ± 0.82	-3.155	0.002
Apolipoprotein (Apo) AI (g/L)	1.28 ± 0.32	1.35 ± 0.31	-3.867	0.000
ApoB (g/L)	0.83 ± 0.21	0.84 ± 0.24	-1.076	0.282
ApoAI/ApoB	1.68 ± 0.78	1.72 ± 0.61	-0.899	0.369

HDL-C, high-density lipoprotein cholesterol; LDL-C, low-density lipoprotein cholesterol. The value of TG was presented as median (interquartile range). The difference between the two ethnic groups was determined by the Wilcoxon-Mann-Whitney test.

### Results of electrophoresis and genotyping

**Figure 1:**
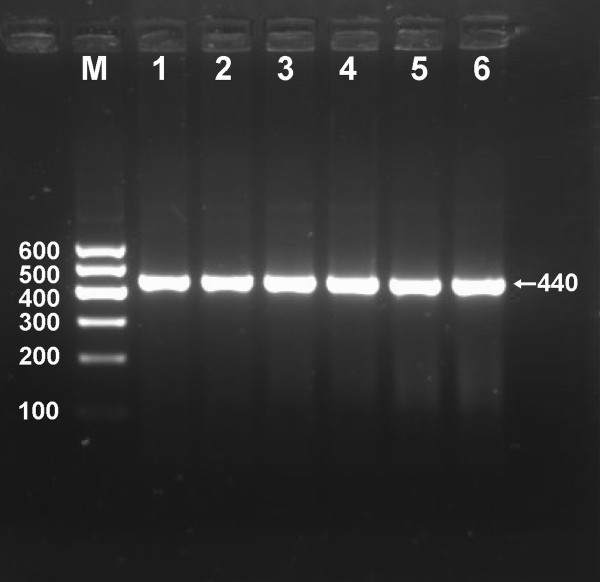
**Electrophoresis of PCR products of the samples**. Lane M, 100 bp marker ladder; lanes 1-6, samples. The 440 bp bands are the target genes.

**Figure 2:**
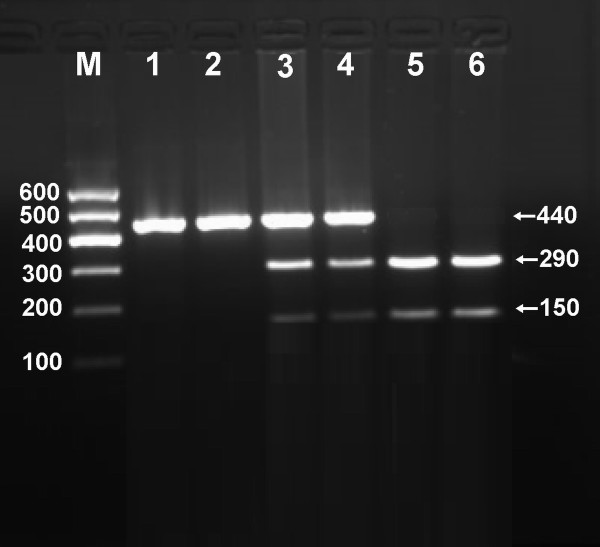
**Genotyping of the PCSK9 E670G polymorphism**. Lane M, 100 bp marker ladder; lanes 1 and 2, GG genotype (440 bp); lanes 3 and 4, AG genotype (440-, 290- and 150-bp); and lanes 5 and 6, AA genotype (290- and 150-bp).

After the genomic DNA of the samples was amplified by PCR and imaged by 2% agarose gel electrophoresis, the purpose gene of 440 bp nucleotide sequences could be seen in all samples (Figure 1). The genotypes identified were named according to the presence or absence of the enzyme restriction sites, when an A to G transversion at 670 locus of the PCSK9 gene. The presence of the cutting site indicates the A allele, while its absence indicates the G allele (cannot be cut). Thus, the GG genotype is homozygote for the absence of the site (band at 440 bp), AG genotype is heterozygote for the absence and presence of the site (bands at 440-, 290- and 150-bp), and AA genotype is homozygote for the presence of the site (bands at 290- and 150-bp; Figure 2). The distribution of genotypes of the SNP followed the Hardy-Weinberg equilibrium.

### Genotypic and allelic frequencies

The genotypic and allelic frequencies of E670G polymorphism in the PCSK9 gene are shown in Table 2. The frequency of A and G alleles was 98.00% and 2.00% in Bai Ku Yao, and 95.20% and 4.80% in Han (*P *< 0.01); respectively. The frequency of AA, AG and GG genotypes was 95.99%, 4.01% and 0% in Bai Ku Yao, and 91.02%, 8.36% and 0.62% in Han (*P *< 0.01); respectively. There was also significant difference in the genotypic and allelic frequencies between normal LDL-C (≤ 3.20 mmol/L) and high LDL-C subgroups (> 3.20 mmol/L, *P *< 0.01) in Bai Ku Yao; and between normal HDL-C (≥ 0.91 mmol/L) and low HDL-C (< 0.91 mmol/L, *P *< 0.05), between normal ApoAI (≥ 1.00 g/L) and low ApoAI (< 1.00 g/L, *P *< 0.05), or between normal ApoAI/ApoB ratio (≥ 1.00) and low ApoAI/ApoB ratio (< 1.00, *P *< 0.01) subgroups in Han.

**Table 2:** Comparison of the genotypic and allelic frequencies of PCSK9 E670G polymorphism between Bai Ku Yao and Han Chinese [n (%)]

Group	n	Genotype			Allele	
		
		AA	AG	GG	A	G
Bai Ku Yao	649	623 (95.99)	26 (4.01)	0 (0.00)	1272 (98.00)	26 (2.00)
Han Chinese	646	588 (91.02)	54 (8.36)	4 (0.62)	1230 (95.20)	62 (4.80)
*χ*^2^	-	14.805			15.447	
*P*	-	0.001			0.001	
Bai Ku Yao						
Male	324	309 (95.37)	15 (4.63)	0 (0.00)	633 (97.69)	15 (2.31)
Female	325	314 (96.62)	11 (3.38)	0 (0.00)	639 (98.92)	11 (1.08)
*χ*^2^	-	0.654			0.641	
*P*	-	0.419			0.423	
Normal TC	549	529 (96.40)	20 (3.60)	0 (0.00)	1078 (98.18)	20 (1.82)
High TC	100	94 (94.00)	6 (6.00)	0 (0.00)	194 (97.00)	6 (3.00)
*χ*^2^	-	1.874			1.197	
*P*	-	0.171			0.274	
Normal TG	550	526 (95.64)	24 (4.36)	0 (0.00)	1076 (97.82)	24 (2.18)
High TG	99	97 (97.98)	2 (2.02)	0 (0.00)	196 (98.99)	2 (1.01)
*χ*^2^	-	1.198			0.653	
*P*	-	0.274			0.419	
Normal HDL-C	460	444 (96.52)	16 (3.48)	0 (0.00)	904 (92.27)	16 (7.73)
Low HDL-C	189	179 (94.71)	10 (5.29)	0 (0.00)	368 (97.35)	10 (2.65)
*χ*^2^	-	1.145			1.121	
*P*	-	0.285			0.290	
Normal LDL-C	488	475 (97.34)	13 (2.66)	0 (0.00)	963 (98.67)	13 (1.33)
High LDL-C	161	148 (91.93)	13 (8.07)	0 (0.00)	309 (95.96)	13 (4.04)
*χ*^2^	-	9.216			9.027	
*P*	-	0.002			0.003	
Normal ApoAI	546	523 (95.79)	23 (4.21)	0 (0.00)	1069 (97.89)	23 (2.11)
Low ApoAI	103	100 (97.09)	3 (2.91)	0 (0.00)	203 (98.54)	3 (1.46)
*χ*^2^	-	0.118			0.115	
*P*	-	0.732			0.734	
Normal ApoB	605	581 (95.87)	25 (4.13)	0 (0.00)	1187 (98.10)	25 (1.90)
High ApoB	43	42 (97.67)	1 (2.33)	0 (0.00)	283 (98.26)	5 (1.74)
*χ*^2^	-	0.338			0.331	
*P*	-	0.561			0.565	
NormalApoAI/ApoB	605	583 (96.36)	22 (3.64)	0 (0.00)	1188 (98.18)	22 (1.82)
Low ApoAI/ApoB	92	84 (91.30)	8 (8.70)	0 (0.00)	84 (95.45)	4 (4.55)
*χ*^2^	-	1.913			1.874	
*P*	-	0.169			0.171	
Han Chinese						
Male	320	288 (90.00)	32 (10.00)	0 (0.00)	608 (95.00)	32 (5.00)
Female	326	300 (92.02)	22 (6.75)	4 (1.23)	626 (96.01)	26 (3.09)
*χ*^2^	-	6.042			0.772	
*P*	-	0.492			0.380	
Normal TC	486	442 (90.95)	40 (8.20)	4 (0.85)	928 (98.56)	44 (1.44)
High TC	160	146 (91.25)	14 (8.75)	0 (0.00)	306 (95.63)	14 (4.37)
*χ*^2^	-	1.357			0.013	
*P*	-	0.507			0.909	
Normal TG	530	480 (90.57)	46 (8.68)	4 (0.75)	1010 (95.28)	50 (4.72)
High TG	116	108 (93.11)	8 (6.89)	0 (0.00)	224 (96.55)	8 (3.45)
*χ*^2^	-	1.305			0.715	
*P*	-	0.521			0.398	
Normal HDL-C	410	366 (89.27)	42 (10.24)	2 (0.49)	776 (94.63)	44 (4.37)
Low HDL-C	236	222 (94.07)	12 (5.08)	2 (0.85)	458 (97.03)	14 (2.97)
*χ*^2^	-	4.222			4.024	
*P*	-	0.039			0.045	
Normal LDL-C	458	416 (90.83)	38 (8.30)	4 (0.87)	874 (95.41)	42 (4.59)
High LDL-C	188	172 (91.49)	16 (8.51)	0 (0.00)	360 (95.75)	16 (4.25)
*χ*^2^	-	1.656			0.068	
*P*	-	0.437			0.795	
Normal ApoAI	594	546 (91.92)	44 (7.41)	4 (0.67)	1136 (95.62)	52 (4.38)
Low ApoAI	52	42 (80.77)	10 (19.23)	0 (0.00)	92 (90.38)	10 (8.62)
*χ*^2^	-	8.999			5.744	
*P*	-	0.011			0.017	
Normal ApoB	580	530 (91.38)	46 (7.93)	4 (0.69)	1060 (95.34)	100 (4.66)
High ApoB	66	58 (87.88)	8 (12.12)	0 (0.00)	124 (93.94)	8 (6.06)
*χ*^2^	-	1.178			1.014	
*P*	-	0.411			0.314	
NormalApoAI/ApoB	624	572 (91.67)	48 (7.67)	4 (0.64)	1192 (95.51)	56 (4.49)
Low ApoAI/ApoB	22	16 (72.73)	6 (27.27)	0 (0.00)	38 (86.36)	6 (13.64)
*χ*^2^	-	10.725			7.787	
*P*	-	0.005			0.005	

Normal TC, TC ≤ 5.17 mmol/L, High TC, TC > 5.17 mmol/L; Normal TG, TG ≤ 1.70 mmol/L, High TG, TG > 1.70 mmol/L; Normal HDL-C, HDL-C ≥ 0.91 mmol/L, Low HDL-C, HDL-C < 0.91 mmol/L; Normal LDL-C, LDL-C ≤ 3.20 mmol/L, High LDL-C, LDL-C > 3.20 mmol/L; Normal ApoAI, ApoAI ≥ 1.00 g/L, Low ApoAI, ApoAI < 1.00 g/L; Normal ApoB, ApoB ≤ 1.14 g/L, High ApoB, ApoB > 1.14 g/L; Normal ApoAI/ApoB, ApoAI/ApoB ≥ 1.00, Low ApoA1/B, ApoAI/ApoB < 1.00.

### Results of sequencing

**Figure 3:**
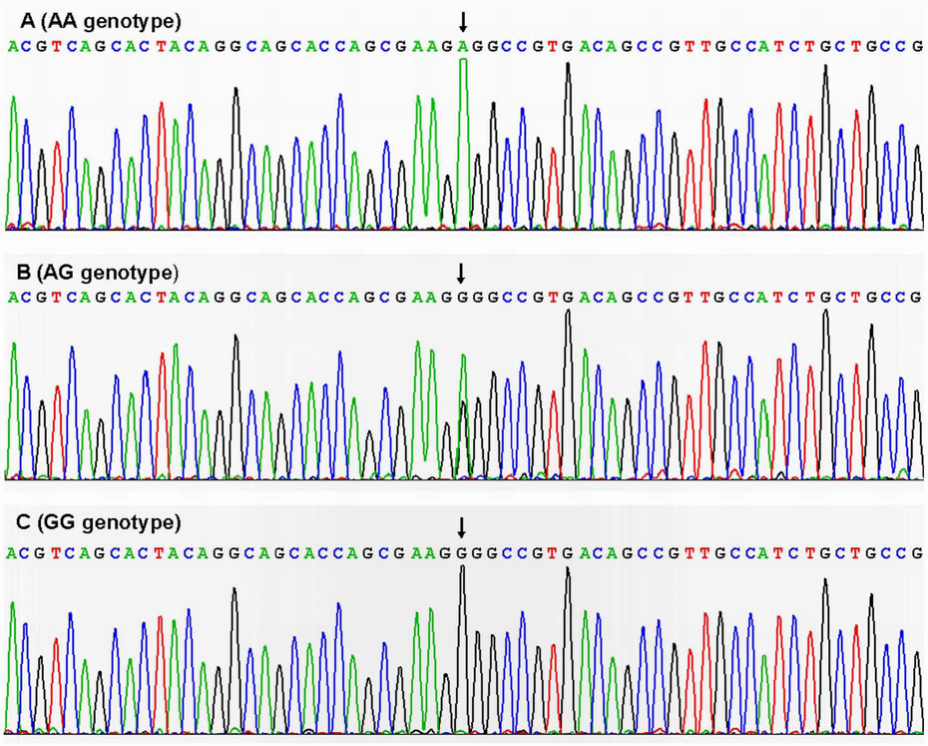
**A part of the nucleotide sequence of the PCSK9 E670G polymorphism**. (A) AA genotype; (B) AG genotype; (C) GG genotype.

The results were shown as AA, AG and GG genotypes by PCR-RFLP, the AA, AG and GG genotypes were also confirmed by sequencing (Figure 3); respectively.

### Genotypes and serum lipid levels

As shown in Table 3, the levels of HDL-C and the ratio of ApoAI to ApoB in Han Chinese but not in Bai Ku Yao were different between the AA and AG/GG genotypes (*P *< 0.05 for each). The G allele carriers had higher serum HDL-C levels and the ratio of ApoAI to ApoB than the G allele noncarriers. When serum lipid parameters in Han were analyzed according to sex, the G allele carriers had higher serum HDL-C and ApoAI levels than the G allele noncarriers in males (*P *< 0.05 for each), whereas the G allele carriers had lower serum ApoB levels and higher the ratio of ApoAI to ApoB than the G allele noncarriers in females (*P *< 0.05 for all).

**Table 3:** Comparison of serum lipid levels among genotypes of Bai Ku Yao and Han Chinese

Genotype	n	TC (mmol/L)	TG (mmol/L)	HDL-C (mmol/L)	LDL-C (mmol/L)	ApoAI (g/L)	ApoB (g/L)	ApoAI/ApoB
Bai Ku Yao								
AA	623	4.27 ± 0.93	0.98(0.67)	1.62 ± 0.40	2.51 ± 0.77	1.28 ± 0.31	0.83 ± 0.21	1.66 ± 0.74
AG/GG	26	4.34 ± 1.08	1.04(0.54)	1.60 ± 0.44	2.58 ± 0.67	1.24 ± 0.35	0.83 ± 0.20	1.59 ± 0.61
*F*	-	0.679	0.089	1.327	0.583	0.042	0.252	0.279
*P*	-	0.410	0.642	0.250	0.445	0.839	0.616	0.597
Male								
AA	309	4.31 ± 1.08	1.09(0.79)	1.63 ± 0.45	2.49 ± 0.91	1.32 ± 0.37	0.82 ± 0.23	1.77 ± 0.94
AG/GG	15	4.46 ± 1.32	1.04(0.62)	1.61 ± 0.46	2.61 ± 0.72	1.26 ± 0.34	0.81 ± 0.22	1.68 ± 0.68
*F*	-	0.742	0.330	0.296	0.792	0.017	0.003	0.003
*P*	-	0.390	0.714	0.587	0.374	0.897	0.958	0.954
Female								
AA	314	4.23 ± 0.76	0.91(0.59)	1.62 ± 0.35	2.54 ± 0.79	1.23 ± 0.24	0.83 ± 0.19	1.56 ± 0.47
AG/GG	11	4.23 ± 0.86	1.05(0.51)	1.60 ± 0.44	2.55 ± 0.90	1.23 ± 0.38	0.85 ± 0.18	1.52 ± 0.56
*F*	-	0.018	0.390	0.181	0.003	0.057	0.221	0.217
*P*	-	0.895	0.696	0.671	0.954	0.811	0.639	0.641
Han Chinese								
AA	588	4.61 ± 1.08	1.01(0.77)	1.74 ± 0.47	2.63 ± 0.82	1.35 ± 0.31	0.84 ± 0.23	1.70 ± 0.59
AG/GG	58	4.79 ± 0.91	1.10(0.66)	1.86 ± 0.55	2.74 ± 0.80	1.41 ± 0.33	0.83 ± 0.26	1.87 ± 0.77
*F*	-	0.607	0.089	3.959	0.332	2.165	0.650	5.394
*P*	-	0.436	0.929	0.047	0.565	0.142	0.420	0.021
Male								
AA	288	4.53 ± 1.07	1.08(0.90)	1.66 ± 0.43	2.58 ± 0.82	1.30 ± 0.29	0.83 ± 0.24	1.68 ± 0.65
AG/GG	32	5.13 ± 0.79	1.13(0.80)	1.86 ± 0.57	2.98 ± 0.65	1.47 ± 0.33	0.94 ± 0.25	1.74 ± 0.82
*F*	-	2.654	0.713	4.365	2.075	4.825	1.464	0.191
*P*	-	0.104	0.476	0.037	0.151	0.029	0.227	0.663
Female								
AA	300	4.69 ± 1.07	0.96(0.68)	1.82 ± 0.48	2.71 ± 0.82	1.39 ± 0.32	0.85 ± 0.22	1.72 ± 0.53
AG/GG	26	4.51 ± 0.92	0.97(0.57)	1.87 ± 0.54	2.56 ± 0.86	1.36 ± 0.31	0.74 ± 0.23	1.97 ± 0.72
*F*	-	0.138	0.190	1.235	0.178	0.130	4.412	6.655
*P*	-	0.710	0.850	0.267	0.674	0.719	0.036	0.010

TC, total cholesterol; TG, triglycerides; HDL-C, high-density lipoprotein cholesterol; LDL-C, low-density lipoprotein cholesterol; ApoAI, apolipoprotein AI; ApoB, apolipoprotein B; ApoAI/ApoB, the ratio of apolipoprotein AI to apolipoprotein B. The value of TG was presented as median (interquartile range). The difference between the genotypes was determined by the Wilcoxon-Mann-Whitney test.

### Relative factors for serum lipid parameters

Multiple linear regression analysis showed that serum HDL-C levels were correlated with genotypes in Han (*P *< 0.05) but not in Bai Ku Yao (Table 4). When multiple linear regression analysis was performed in the males and females in both ethnic groups; respectively, we showed that serum HDL-C and ApoAI levels in Han but not in Bai Ku Yao were correlated with genotypes (*P *< 0.05) in males, and serum ApoB levels and the ratio of ApoAI to ApoB were associated with genotypes (*P *< 0.05, Table 5) in females. Serum lipid parameters were also correlated with sex, age, BMI, alcohol consumption, cigarette smoking, and blood pressure in both ethnic groups (*P *< 0.05-0.001, Tables 4 and 5).

**Table 4:** Correlative factors for serum lipid parameters between Bai Ku Yao and Han Chinese

Lipid parameter	Relative factor	Unstandardized coefficient	Std. error	Standardized coefficient	*t*	*P*
Bai plus Han						
TC	Diastolic blood pressure	0.020	0.003	0.205	7.026	0.000
	Body mass index	0.060	0.009	0.190	6.812	0.000
	Age	0.108	0.017	0.180	6.544	0.000
	Sex	0.115	0.053	0.058	2.171	0.030
	Ethnic group	-0.332	0.052	-0.163	-6.355	0.000
TG	Body mass index	0.063	0.011	0.162	5.599	0.000
	Sex	-0.236	0.073	-0.097	-3.208	0.001
	Alcohol consumption	0.146	0.047	0.094	3.129	0.002
	Ethnic group	-0.144	0.065	-0.060	-2.217	0.027
HDL-C	Age	0.064	0.008	0.237	8.187	0.000
	Ethnic group	-0.128	0.024	-0.144	-5.295	0.000
	Body mass index	-0.019	0.004	-0.132	-4.561	0.000
	Sex	0.137	0.027	0.155	5.101	0.000
	Alcohol consumption	0.077	0.017	0.136	4.463	0.000
	Genotype	0.126	0.049	0.069	2.554	0.011
	Diastolic blood pressure	0.003	0.001	0.066	2.189	0.029
LDL-C	Body mass index	0.055	0.007	0.226	8.042	0.000
	Age	0.079	0.013	0.171	6.087	0.000
	Diastolic blood pressure	0.011	0.002	0.149	5.138	0.000
	Alcohol consumption	-0.132	0.026	-0.136	-4.978	0.000
	Ethnic group	-0.107	0.042	-0.067	-2.528	0.012
ApoAI	Age	0.050	0.006	0.267	8.814	0.000
	Alcohol consumption	0.074	0.012	0.186	6.273	0.000
	Sex	0.056	0.018	0.091	3.093	0.002
	Ethnic group	-0.078	0.017	0.123	-4.651	0.000
	Diastolic blood pressure	0.001	0.001	0.079	2.629	0.009
ApoB	Body mass index	0.017	0.002	0.243	8.716	0.000
	Systolic Blood pressure	0.004	0.001	0.176	6.041	0.000
	Age	0.020	0.004	0.148	5.392	0.000
Bai Ku Yao						
TC	Body mass index	0.076	0.014	0.211	5.273	0.000
	Sex	0.081	0.024	0.140	3.431	0.000
	Systolic blood pressure	0.008	0.004	0.088	2.139	0.033
TG	Sex	-0.478	0.118	-0.211	-4.060	0.001
	Body mass index	0.069	0.020	0.141	3.498	0.001
	Alcohol consumption	0.176	0.063	0.133	2.816	0.005
	Cigarette smoking	-0.179	0.073	-0.128	-2.455	0.014
HDL-C	Age	0.052	0.011	0.192	4.622	0.000
	Alcohol consumption	0.077	0.022	0.169	3.580	0.000
	Sex	0.071	0.036	0.091	1.998	0.046
LDL-C	Body mass index	0.066	0.012	0.223	5.560	0.000
	Sex	0.064	0.020	0.137	3.264	0.001
	Alcohol consumption	-0.107	0.033	-0.135	-3.272	0.001
	Diastolic blood pressure	0.007	0.003	0.091	2.192	0.029
ApoAI	Alcohol consumption	0.090	0.014	0.253	6.313	0.000
	Age	0.040	0.008	0.192	4.783	0.000
ApoB	Body mass index	0.019	0.004	0.217	5.374	0.000
	Age	0.017	0.006	0.117	2.772	0.006
	Diastolic blood pressure	0.002	0.001	0.095	2.286	0.023
	Alcohol consumption	-0.023	0.010	-0.094	-2.266	0.024
ApoAI/ApoB	Sex	-0.234	0.060	-0.150	-3.903	0.000
	Body mass index	-0.042	0.013	-0.122	-3.143	0.002
	Age	0.057	0.021	0.106	2.738	0.006
Han Chinese						
TC	Diastolic blood pressure	0.028	0.004	0.283	7.042	0.000
	Age	0.111	0.022	0.186	4.95	0.000
	Body mass index	0.050	0.011	0.172	4.464	0.000
	Sex	0.354	0.088	0.165	4.022	0.000
	Cigarette smoking	0.110	0.053	0.086	2.082	0.038
TG	Diastolic blood pressure	0.020	0.005	0.176	4.174	0.000
	Body mass index	0.059	0.014	0.171	4.181	0.000
	Cigarette smoking	0.148	0.063	0.097	2.357	0.019
	Alcohol consumption	0.156	0.076	0.085	2.045	0.041
HDL-C	Age	0.067	0.011	0.253	6.404	0.000
	Sex	0.216	0.038	0.226	5.605	0.000
	Alcohol consumption	0.104	0.028	0.151	3.746	0.000
	Body mass index	-0.024	0.005	-0.187	-4.586	0.000
	Diastolic blood pressure	0.005	0.002	0.114	2.662	0.008
	Genotype	0.148	0.062	0.088	2.384	0.017
LDL-C	Body mass index	0.050	0.009	0.224	5.616	0.000
	Age	0.077	0.018	0.170	4.379	0.000
	Diastolic blood pressure	0.015	0.003	0.200	4.813	0.000
	Alcohol consumption	-0.128	0.048	-0.108	-2.67	0.008
	Sex	0.200	0.071	0.122	2.835	0.005
	Cigarette smoking	0.088	0.043	0.09	2.053	0.040
ApoAI	Age	0.058	0.007	0.336	8.923	0.000
	Sex	0.131	0.024	0.211	5.458	0.000
	Alcohol consumption	0.075	0.017	0.168	4.333	0.000
	Diastolic blood pressure	0.003	0.001	0.113	2.940	0.003
ApoB	Body mass index	0.016	0.002	0.247	6.400	0.000
	Diastolic blood pressure	0.005	0.001	0.220	5.633	0.000
	Age	0.025	0.005	0.188	5.082	0.000
ApoAI/ApoB	Body mass index	-0.043	0.007	-0.256	-6.491	0.000
	Age	0.045	0.013	0.133	3.384	0.001

TC, total cholesterol; TG, triglyceride; HDL-C, high-density lipoprotein cholesterol; LDL-C, low-density lipoprotein cholesterol; ApoAI, apolipoprotein AI; ApoB, apolipoprotein B.

**Table 5:** Correlative factors for serum lipid parameters between males and females in both ethnic groups

Lipid parameter	Relative factor	Unstandardized coefficient	Std. error	Standardized coefficient	*t*	*P*
Bai Ku Yao						
Male						
TC	Body mass index	0.130	0.027	0.267	4.869	0.000
	Age	0.105	0.041	0.139	2.539	0.012
TG	Body mass index	0.128	0.036	0.197	3.516	0.001
HDL-C	Age	0.064	0.018	0.204	3.563	0.000
	Body mass index	-0.031	0.011	-0.155	-2.870	0.004
	Alcohol comsumption	0.117	0.027	0.249	4.325	0.000
LDL-C	Body mass index	0.111	0.022	0.276	5.022	0.000
ApoAI	Age	0.047	0.014	0.185	3.281	0.001
	Alcohol comsumption	0.117	0.022	0.304	5.405	0.000
ApoB	Body mass index	0.032	0.006	0.312	5.741	0.000
ApoAI/ApoB	Body mass index	-0.090	0.023	-0.219	-3.947	0.000
	Pulse pressure	0.009	0.004	0.113	2.044	0.042
	Alcohol comsumption	0.223	0.054	0.230	4.107	0.000
Female						
TC	Body mass index	0.040	0.018	0.124	2.282	0.023
	Systolic blood pressure	0.009	0.003	0.187	3.435	0.001
TG	Alcohol comsumption	0.297	0.070	0.231	4.261	0.000
HDL-C	Age	0.045	0.013	0.183	3.340	0.001
LDL-C	Body mass index	0.032	0.014	0.126	2.327	0.021
	Age	0.050	0.025	0.119	2.034	0.043
	Systolic blood pressure	0.006	0.002	0.161	2.731	0.007
ApoAI	Age	0.038	0.009	0.217	3.988	0.000
ApoB	Body mass index	0.010	0.004	0.127	2.338	0.020
	Systolic blood pressure	0.002	0.001	0.202	3.716	0.000
Han Chinese						
Male						
TC	Diastolic blood pressure	0.030	0.005	0.319	5.736	0.000
	Body mass index	0.046	0.014	0.174	3.259	0.001
	Age	0.093	0.031	0.156	2.996	0.003
TG	Body mass index	0.103	0.020	0.293	5.223	0.000
	Diastolic blood pressure	0.016	0.007	0.127	2.230	0.026
	Alcohol comsumption	0.192	0.087	0.116	2.199	0.029
HDL-C	Age	0.046	0.014	0.187	3.198	0.002
	Body mass index	-0.032	0.006	-0.295	-5.471	0.000
	Alcohol comsumption	0.105	0.027	0.202	3.882	0.000
	Systolic blood pressure	0.005	0.002	0.176	2.947	0.003
	Genotype	0.192	0.084	0.117	2.277	0.023
LDL-C	Diastolic blood pressure	0.017	0.004	0.247	4.351	0.000
	Body mass index	0.039	0.011	0.195	3.440	0.001
ApoAI	Age	0.046	0.009	0.288	5.179	0.000
	Alcohol comsumption	0.075	0.017	0.220	4.448	0.000
	Systolic blood pressure	0.004	0.001	0.209	3.682	0.000
	Body mass index	-0.011	0.004	-0.156	-3.030	0.003
	Genotype	0.128	0.052	0.120	2.444	0.015
ApoB	Diastolic blood pressure	0.006	0.001	0.279	5.154	0.000
	Body mass index	0.017	0.003	0.278	5.141	0.000
ApoAI/ApoB	Body mass index	-0.047	0.009	-0.289	-5.378	0.000
	Pulse pressure	0.009	0.003	0.157	2.813	0.005
	Age	0.056	0.021	0.153	2.700	0.007
Female						
TC	Diastolic blood pressure	0.025	0.006	0.234	4.267	0.000
	Body mass index	0.056	0.019	0.168	3.018	0.003
	Age	0.115	0.032	0.195	3.623	0.000
TG	Diastolic blood pressure	0.024	0.006	0.225	4.158	0.000
HDL-C	Age	0.071	0.015	0.263	4.906	0.000
LDL-C	Diastolic blood pressure	0.015	0.005	0.178	3.263	0.001
	Body mass index	0.062	0.014	0.239	4.310	0.000
	Age	0.085	0.025	0.185	3.443	0.001
ApoAI	Age	0.067	0.009	0.373	7.253	0.000
	Alcohol comsumption	0.106	0.052	0.105	2.042	0.042
ApoB	Diastolic blood pressure	0.004	0.001	0.200	3.755	0.000
	Body mass index	0.016	0.004	0.223	4.130	0.000
	Age	0.027	0.007	0.219	4.160	0.000
	Genotype	-0.075	0.037	-0.099	-2.036	0.043
ApoAI/ApoB	Body mass index	-0.032	0.010	-0.181	-3.348	0.001
	Genotype	0.249	0.101	0.133	2.457	0.015

TC, total cholesterol; TG, triglyceride; HDL-C, high-density lipoprotein cholesterol; LDL-C, low-density lipoprotein cholesterol; ApoAI, apolipoprotein AI; ApoB, apolipoprotein B.

## Discussion

We showed that the levels of serum TC, HDL-C, LDL-C and ApoAI were lower in Bai Ku Yao than in Han Chinese. There was no significant difference in the serum levels of TG, ApoB and the ratio of ApoAI to ApoB between the two ethnic groups. It is well known that dyslipidemia is a multifactorial origin, including environmental factors such as demographics, diet, alcohol consumption, cigarette smoking, obesity, exercise, hypertension; genetic factors such as variants in genes coding for proteins; and their interactions [7-13]. Bai Ku Yao is a special and isolated subgroup of the Yao minority in China. Their dietary habits and lifestyle are very special. In addition, strict intra-ethnic marriages have been performed in this ethnic subgroup from time immemorial. Therefore, we believe that some hereditary characteristics and genotypes of lipid metabolism-related genes in this population may be different from those in Han Chinese [9-11].

The frequency spectrum of E670G mutation varied significantly among different races/ethnicities. For example, the frequency of G allele is rare in whites but present in approximately 24.8% of blacks [45]. Kotowski *et al*. [37] also reported that the minor-allele frequency (670G) in the Dallas Heart Study (DHS) was 3.6% in whites, 4.2% in Hispanics, and 26.0% in blacks. The frequency of the G allele in patients selected from Universitätsklinikum Hamburg-Eppendorf Martinistrasse, Hamburg, Germany was 5% (AA 458, AG 45, GG 3) [39] which lies between that observed in the TexGen population, 4.4% (AA 291, AG 28, GG 0) and that reported for the Lipoprotein Coronary Atherosclerosis Study (LCAS), 7.4% (AA 324, AG 41, GG 7) by Chen *et al*. [38] in their original study. There was no statistical significant difference in the frequency of the G allele in patients with LDL-C below the 50th percentile for age and sex, 4.4% (AA 93, AG 9, GG 0), those with LDL-C between the 50th and 95th percentiles, 6.4% (AA 160, AG 20, GG 1) and those with LDL-C above the 95th percentile, 6.4% (AA 205, AG 26, GG 2) [39]. The 670G carrier in Chinese Taiwanese was identified less frequently in patients with CAD than in controls (9.9% vs. 11.9%), but the difference was not significant in a multivariable logistic regression analysis [41]. Two previous studies, however, showed that the G allele frequency, 3.4% in healthy U.K. men and patients with clinically defined definite familial hypercholesterolaemia was not associated with any significant effects on plasma lipid levels or CAD risk [43], and 6.0% in Prospective Study of Pravastatin in the Elderly at Risk (PROSPER) was no significant relationships with baseline LDL-C, response to pravastatin, or vascular disease risk being observed [44]. In the present study, we showed that the frequency of G allele of E670G polymorphism in the PCSK9 gene was lower in Bai Ku Yao (2.00%) than in Han Chinese (4.80%). The frequency of AG/GG genotype was also lower in Bai Ku Yao than in Han. There was also significant difference in the genotypic and allelic frequencies between normal and high LDL-C subgroups in Bai Ku Yao; and between normal and low HDL-C, between normal and low ApoAI, or between normal and low ApoAI/ApoB ratio subgroups in Han. These results indicate that the prevalence of the G allele variation of E670G in the PCSK9 gene may have an ethnic specificity.

The association of E670G polymorphism in the PCSK9 gene and serum lipid levels is still controversial in several previous studies from ethnically diverse populations. Chen *et al*. [38] have reported that haplotype structure analysis identified E670G as the determinant variant among a black population, exerting a dose effect (GG > EG > EE) and accounting for 3.5% of plasma LDL-C variability (*F *= 14.6, *P *< 0.001). Plasma TC, ApoB, and lipoprotein (a) levels were also associated with the E670G variant. Distributions of the E670G genotypes in an independent normolipidemic and the hyperlipidemic subjects in the LCAS population were significantly different (*F *= 7.2, *P *= 0.027). Evans and Beil [39] found that the PCSK9 E670G polymorphism in a European population was associated with increased LDL-C in men but not in women. Norata *et al*. [40] also showed that the 670G carriers were associated with increased plasma TC, LDL-C, and ApoB levels in the general population. The intima media thickness (IMT) was significantly increased in 670G carriers compared to individuals homozygous for the A allele. The presence of the 670G allele was also significantly associated with a greater progression of IMT compared to 670AA subjects. Contradictory to these previous results, Hsu *et al*. [41] showed a significantly lower level of LDL-C in 670G carriers in Chinese Taiwanese than in non-carriers (2.78 ± 0.82 mmol/L vs. 3.02 ± 0.85 mmol/L; *P *= 0.029) among 614 unrelated controls, after adjusting for age, gender, smoking, hypertension, diabetes mellitus, BMI, and use of lipid-lowering agents. However, the association between the E670G polymorphism in the PCSK9 gene and plasma LDL-C levels was not confirmed in other studies [37,43-45]. In the present study, we showed that the G allele carriers in Han but not in Bai Ku Yao had higher serum HDL-C levels and the ratio of ApoAI to ApoB than the G allele noncarriers. When serum lipid parameters in Han were analyzed according to sex, the G allele carriers had higher serum HDL-C and ApoAI levels than the G allele noncarriers in males, whereas the G allele carriers had lower serum ApoB levels and higher the ratio of ApoAI to ApoB than the G allele noncarriers in females. Multiple linear regression analysis showed that the levels of serum HDL-C and ApoAI in Han were correlated with genotypes in males, and the levels of ApoB and the ratio of ApoAI to ApoB were associated with genotypes in females. Although we showed that the levels of serum LDL-C were slightly higher in the subjects with AG/GG genotype than those with AA genotype, the difference did not reach statistical significance. These results suggest that the PCSK9 E670G polymorphism is mainly associated with some serum lipid parameters in the Han population. The reason for this discrepancy is most likely due to racial and ethnic differences among these studies. The study sample of Evans and Beil was men of European origin, the majority (84%) of Chen's sample was American whites, and the sample of Huang *et al*. [45] was restricted to blacks [124/1750 (7%), because only 3 participants of 1828 whites were found to have this PCSK9 genetic variant. In contrast, our results regarding the E670G polymorphism are restricted to two Chinese populations, Han and Bai Ku Yao. Our study sample is also not different from that of Hsu *et al*. [41], which included 202 CAD patients and 614 unrelated controls (Chinese Taiwanese).

It is well known that dietary patterns, like the Mediterranean diet, are strongly related with blood lipids levels, as well as with the prevalence and the management of dyslipidemia [50]. We slao showed that serum lipid parameters were correlated with age, sex, alcohol consumption, cigarette smoking, BMI, and blood pressure in this study. These data suggest that the environmental factors also play an important role in determining serum lipid levels in our populations [12,13]. The diet and lifestyle were different between the two ethnic groups. Corn was the staple food and rice, soy, buckwheat, sweet potato, and pumpkin products were the subsidiary foods in Bai Ku Yao. Approximately 90% of the beverages were corn wine and rum. The alcohol content is about 15% (v/v). They are also accustomed to drink hempseed soup and eat hempseed products. In contrast, rice was the staple food and corn, broomcorn, potato, and taro products were the subsidiary foods in Han. About 90% of the beverage was rice wine. The content of alcohol is about 30% (v/v). The staple and subsidiary foods are more favorable for serum lipid profiles in Bai Ku Yao than in Han. Corn contains abundant dietary fiber and plant protein [51]. Consumption of dietary fiber, specifically the soluble type, such as pectins and guar gum can decrease serum TC levels [52,53]. Levels of total cholesterol (TC) and low-density lipoprotein cholesterol (LDL-C) and the LDL/HDL ratio in subjects with moderate hypercholesterolemia were significantly reduced during a mixture of dietary fiber (guar gum, pectin, soy, pea, corn bran) treatment. The mean percentage reductions from baseline after 51 weeks of treatment were approximately 5% for TC, 9% for LDL-C, and 11% for the LDL/HDL ratio. There were no significant effects on the levels of either triglycerides or high-density lipoprotein cholesterol (HDL-C) [53]. Plant protein might promote the transportation and excretion of free cholesterol. Dietary soy protein has well-documented beneficial effects on serum lipid concentrations [54,55]. Soy protein intake is effective in reducing TC by 9.3%, LDL-C by 12.9%, and TG by 10.5% and in increasing HDL-C by 2.4%. Furthermore, the changes in serum TC and LDL-C concentrations were directly related to the initial serum TC concentration [54]. A meta-analyse including 10 studies showed that feeding daily 36 g soy protein with 52 mg soy-associated isoflavones on average decreased LDL-C by -0.17 ± 0.04 mmol/L and increased HDL-C by 0.03 ± 0.01 mmol/L [55]. Buckwheat protein product has a potent hypocholesterolemic activity [56,57]. Son *et al*. [57] found that plasma lipid profiles in rats differed significantly according to grain combination. The levels of TG in the adlay, buckwheat and waxy barley groups were significantly lower than those in the white rice group. The buckwheat and waxy barley groups showed lower levels of TC and LDL-C, and higher HDL-C than the white rice group. Ingestion of 4 g/day caiapo (the extract of the white-skinned sweet potato Ipomoea batatas) for 6 weeks has been found to reduce plasma TC, LDL-C levels in type 2 diabetic patients previously treated by diet alone [58]. Replacing two thirds of staple food with yam (Dioscorea alata) for 30 days in healthy postmenopausal women can significantly decreased plasma cholesterol concentration by 5.9%, prolonged the lag time of LDL lipoprotein oxidation by 5.8%, and decreased urinary isoprostane levels by 42% [59]. Adaramoye *et al*. [60] reported that supplemented diets containing 3% and 6% telfairia occidentalis (fluted pumpkin) in rats decreased plasma and postmitochondrial supernatant fraction (PMF) cholesterol levels by 20% and 30% and by 30% and 45%, respectively; decreased the cholesterol-induced increase in plasma and PMF LDL-C levels by 24% and 48% and by 28% and 52%, respectively; and decreased plasma and PMF lipid peroxidation by 24% and 20% and by 42% and 21%, respectively. Dietary hempseed is a rich source of polyunsaturated fatty acids (PUFAs). Hempseed-supplemented diet in animals displayed elevated plasma levels of PUFAs and a prominent enhancement in gamma-linolenic acid levels. When hempseed is added to a cholesterol-enriched diet, cholesterol-induced platelet aggregation returns to control levels [61,62]. This normalization may be partly due to increased levels of plasma gamma-linolenic acid [61]. In addition, several experimental and clinical studies have demonstrated that dietary hempseed or hempseed oil can decrease TC, TG and LDL-C levels [63-65], inhibit lipid peroxidation [66], and reduce atherogenic index [67].

## Conclusion

The present study shows that the frequency of G allele of E670G polymorphism in the PCSK9 gene was lower in Bai Ku Yao than in Han. There was also significant difference in the genotypic and allelic frequencies between normal and high LDL-C subgroups in Bai Ku Yao; and between normal and low HDL-C, between normal and low ApoAI, or between normal and low ApoAI/ApoB ratio subgroups in Han. The G allele carriers in Han had higher serum HDL-C and ApoAI levels than the G allele noncarriers in males, whereas the G allele carriers had lower serum ApoB levels and higher the ratio of ApoAI to ApoB than the G allele noncarriers in females. Serum HDL-C and ApoAI levels in Han were correlated with genotypes in males, and serum ApoB levels and the ratio of ApoAI to ApoB were associated with genotypes in females. These results suggest that the PCSK9 E670G polymorphism is mainly associated with some serum lipid parameters in the Han population. The G allele carriers had higher serum HDL-C and ApoAI levels in males, and lower serum ApoB levels and higher the ApoAI/ApoB ratio in females than the G allele noncarriers.

## Competing interests

The authors declare that they have no competing interests.

## Authors' contributions

LHHA participated in the design, undertook genotyping, and helped to draft the manuscript. RXY conceived the study, participated in the design, carried out the epidemiological survey, collected the samples, and drafted the manuscript. LM, XJH, TTY, XLC, DFW, and QL collaborated to the genotyping. SLP and JZW carried out the epidemiological survey, collected the samples, and helped to carry out the genotyping. All authors read and approved the final manuscript.
